# Management of Allogeneic Stem Cell Transplantation for High-Risk AML following SARS-CoV-2 Associated Pancytopenia with Marked Bone Marrow Biopsy Alterations

**DOI:** 10.1155/2021/8843063

**Published:** 2021-01-21

**Authors:** Maria Kaparou, Zbigniew Rudzki, Hannah Giles, Vidhya Murthy, Swathy Srinath, Rebecca Lloyd, Maria Zahid Ahmed, Beena Salhan, Saleena Chauhan, Bhuvan Kishore, Richard Lovell, Claire Horgan, Shankara Paneesha, Evgenia Xenou, Anand Lokare, Joanne Ewing, Hansini Dassanayake, Emmanouil Nikolousis, Alexandros Kanellopoulos

**Affiliations:** University Hospitals Birmingham NHS Foundation Trust, London, UK

## Abstract

The present study describes a patient aged 70 with very high-risk AML who successfully received a nonmyeloablative matched unrelated donor allograft shortly following SARS-CoV-2 infection, which manifested with mild cough, interstitial abnormalities on chest CT, and pancytopenia with profound bone marrow biopsy histological alterations. In parallel, our study provides bone marrow biopsy data in a series of contemporary patients with serious haematological diseases who had a bone marrow biopsy performed within two weeks of PCR confirmation of SARS-CoV-2 infection. This study is notable because there are no published data describing the bone marrow biopsy changes observed in patients with haematological malignancies and SARS-CoV-2 infection. Finally, it is suggested that nonmyeloablative hematopoietic stem cell transplantation for very high-risk haematological malignancies can be successfully performed following recovery from SARS-CoV-2 infection.

## 1. Case Report

Corona virus disease 2019 (COVID-19), which is caused by the severe acute respiratory syndrome corona virus 2 (SARS-CoV-2), has infected millions of people all over the world and over one million deaths reported [1].

Individuals infected by SARS-CoV-2 experience a broad spectrum of symptoms, ranging from mild respiratory symptoms to severe respiratory and multiorgan failure [2]. Pathological findings precipitated by the SARS-CoV-2 virus are under intense study, but there is a paucity of data with regards to bone marrow involvement [3]. Herein, we describe the case of a 70-year-old female patient with acute myeloid leukaemia (AML) who had clinically mild COVID-19 infection pretransplant, which resulted in new onset cytopenias and novel bone marrow morphological changes in the absence of disease relapse.

The patient was diagnosed with AML with myelodysplastic changes based on myelodysplastic syndrome- (MDS-) related cytogenetic features but with no morphological evidence of dysplasia in December 2019. She had no significant comorbidities. The immunophenotype of the neoplastic cells at diagnosis was CD34+/CD117+/CD13+/HLA-DR+/nTdt+/CD33-/MPO weak. The karyotype and next generation sequencing panel showed an abnormal karyotype with trisomy13/add(17p) and clinically significant variants in ASXL1, RUNX1, IDH2, and BCOR, respectively.

The patient was treated with two cycles of CPX-351 (liposomal cytarabine/daunorubicin), which were completed in February 2020 and resulted in complete remission. Bone marrow biopsy after cycle two of induction chemotherapy showed prominent postchemotherapy damage and dysplastic megakaryocytes but no excess of blasts, consistent with a dysplastic remission. Karyotype was normal, consistent with cytogenetic remission. The complete blood count (CBC) at that time was Hb 85 g/L, WBC 4.34 × 10^9^/L, neutrophils 1.24 × 10^9^/L, and platelets 85 × 10^9^/L.

Based on the very high-risk genetic profile and taking into account patient fitness and preference, a fully matched volunteer unrelated donor (MUD) hematopoietic stem cell transplant, conditioned with fludarabine/cyclophosphamide/total body irradiation 2 Gy with post-transplant cyclophosphamide/tacrolimus/mycophenolate mofetil for primary graft versus host disease prophylaxis, was deployed for consolidation due to the high-relapse risk and the low rates of chronic graft-versus-host disease observed with this T-replete nonmyeloablative conditioning regimen [4].

**Figure 1:**
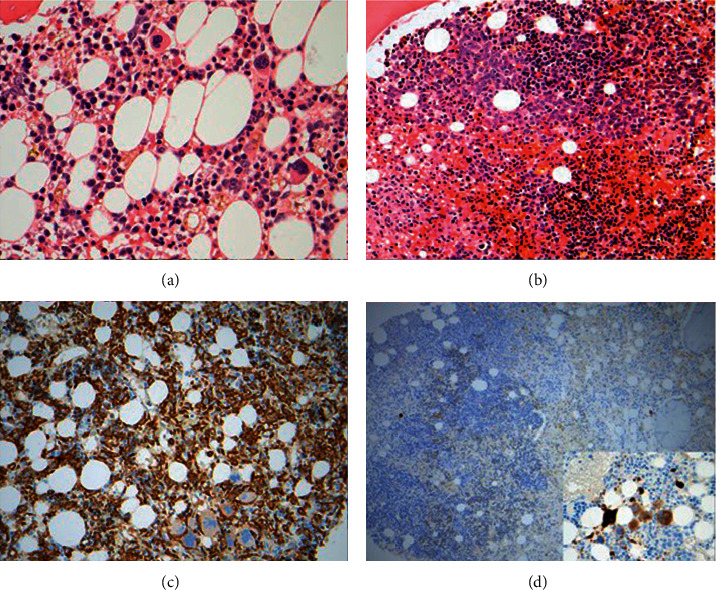
Bone marrow biopsy findings before and after COVID-19 diagnosis. (a) Before SARS-CoV-2 detection, all three main hematopoietic lineages were visible. There was no significant dysplasia. Erythroid cells were relatively scant and scattered. There was subtle stromal damage. (b) Following SARS-CoV-2 infection, prominent erythroid hyperplasia and signs of stromal damage were observed. Large clusters of erythroid cells were locally synchronised at the same maturational stage (“stress erythropoiesis”). Proerythroblasts showed megaloblastoid features. Erythroid cells approached the trabecular bone. (c) An excess of stromal histiocytes on CD163 immunostaining after SARS-CoV-2 infection. (d) On CD61 immunostaining, profound megakaryocytic deficit and atypia were illustrated after SARS-CoV-2 infection.

The patient had a screening nasopharyngeal swab for SARS-CoV-2 before the planned admission date for the transplant, which was reported as positive. The transplant was therefore postponed. Apart from mild fatigue and mild dry cough, the patient did not report significant symptoms. CRP was only mildly elevated, peak level recorded 39 mg/L. Over the next two weeks, the patient became more pancytopenic and required transfusion support with packed red cells and platelets (CBC at the end of April: Hb 67 g/L, WBC 2.30 × 10^9^/L, neutrophils 0.81 × 10^9^/L, and platelets 7 × 10^9^/L). Repeat bone marrow biopsy showed no evidence of relapse, but accentuated myelodysplasia-related findings including striking erythroid hyperplasia, megaloblastic features (folate 2.4 *µ*g/L and vitamin B12 371 ng/L), extreme “stress-type” arrangement of erythroid cells, myeloid suppression, reduced numbers of megakaryocytes with megakaryocytic atypia, and increased histiocytes (confirmed on CD163 and CD68/PGM1 immunostaining) in conjunction with other features of stromal damage, consistent with very pronounced toxic-type injury (Figure 1). The aspirate showed abundant foamy macrophages with markedly reduced megakaryocytes, some haemophagocytosis, and mild erythroid dysplasia. Liver function tests confirmed elevated bilirubin and ferritin at 31 *µ*mol/L and 1854 ng/mL, respectively, with normal enzymes. The patient was afebrile and did not have any organomegaly. The karyotype was normal. Serology was negative for HIV, hepatitis B/C/E, and oarvovirus, whereas the patient was negative for EBV and CMV by PCR. PT was only mildly prolonged at 15 seconds and APTT was never prolonged; D-dimer and fibrinogen were not measured. High resolution CT scan of the chest demonstrated bilateral air space opacities, with ground glass changes and interlobular septal thickening in the right lower lobe. The patient underwent bronchoalveolar lavage which did not isolate any other viral, fungal, or bacterial pathogen. In all, there were neither respiratory sequelae (other than the mild cough), nor any need to treat with remdesivir or steroids, as repeat SARS-CoV-2 nasopharyngeal swabs were negative and there was improvement in the findings in the follow-up high resolution CT scan of the chest. The patient therefore proceeded with the planned nonmyeloablative MUD allogeneic stem cell transplant. The peritransplant period was complicated by a single episode of culture negative febrile neutropenia and delayed engraftment (neutrophils-platelets engrafted on days +25 and +45, respectively). The patient remains well, disease-free (100% chimerism), without infective concerns, or active GvHD as of the time of writing this report, on day +180 out from transplant.

## 2. Discussion

This case describes an individual who was incidentally diagnosed with SARS-COV-2 infection before a planned bone marrow transplant for high-risk AML with MDS-related changes. At the time of SARS-CoV-2 infection, the patient was in complete remission with near normal counts, but the virus triggered new onset transfusion-dependent cytopenias. The transplant was therefore deferred by four weeks. Bone marrow biopsy two weeks after SARS-COV-2 infection diagnosis revealed marked changes (i.e., exacerbated dysplasia in hematopoietic cells, abundant histiocytes, and toxic stromal changes). To date, there are no data from patient series with regards to SARS-CoV-2-induced bone marrow biopsy changes although there have been scant data about bone marrow haemophagocytosis in bone marrow aspirates in patients with severe SARS-CoV-2 infection [5].

The haematological manifestations in patients with symptomatic SARS-CoV-2 infection have been widely reported and include leukopenia with lymphopenia and mild thrombocytopenia. However, the exact mechanisms causing these cytopenias are unclear and might involve both peripheral destruction/consumption and medullary dysfunction [6].

Cytopenias may be caused by a reduction in CD34+ cells, which may occur due to a variety of mechanisms. The SARS-CoV-2 virus can bind to the ACE2 receptor on the surface of hematopoietic cells and be internalized into CD34+ cells, resulting in viral replication and apoptosis. This process may be facilitated by CD13 and CD66a (EACAMIa) receptors, which are present on the surface of human bone marrow CD34+ cells. It has been also proposed that autoantibodies targeting CD34+ bone marrow cells may be produced in some individuals with SARS-CoV-2 infection [7, 8]. SARS-CoV-2 may also affect bone marrow stromal cells, leading to an altered bone marrow microenvironment.

Patients with severe SARS-CoV-2 infection experience an excessive inflammatory response characterized by high levels of cytokines, profound lymphopenia, and substantial mononuclear cell infiltration in the lungs, heart, spleen lymph nodes, and kidneys [9]. Destruction of bone marrow progenitor cells by the cytokine storm could overwhelm the rate of blood cell production, resulting in cytopenias [10]. Macrophages are key players as they can be evaded directly by the virus and activated by inflammatory signals, such as type I interferon, with recruitment to inflamed tissues via chemokine receptor CCR-2 [11, 12]. This explains the accumulation of histiocytes and stromal abnormalities observed in this patient's bone marrow biopsy after infection with the SARS-CoV-2 virus. Similar changes also were observed in two other patients in our centre who were diagnosed with COVID-19 as discussed in Table 1 (patient number six who died of COVID-19 after BEAM-autologous stem cell for nodular lymphocyte predominant Hodgkin lymphoma and case two with newly diagnosed blastoid plasma cell myeloma who also developed severe thrombocytopenia amidst velcade/thalidomide/dexamethasone induction treatment). The remaining patients described in Table 1 had newly diagnosed haematological disorders and the causative role of the SARS-CoV-2 infection in their abnormal bone marrow histology is felt less likely.

The patient described in this report had been diagnosed with high-risk AML with MDS-related changes The bone marrow microenvironment in MDS is characterized by higher levels of inflammatory cytokines and upregulation of cell surface receptors for proinflammatory molecules [13], such as TGF-beta, IL-1-beta and, danger-associated molecular pattern (DAMP) proteins. Therefore, the “dysplastic” hematopoietic progenitor cells and their bone marrow niche inflicted severe dysfunction during an otherwise mild COVID-19.

The immunocompromised nature of patients with haematological malignancies, including patients undergoing allogeneic stem cell transplantation, could have a negative impact on clearance of the SARS-CoV-2 virus. On the other hand, it might play an important role in later phases of the disease, by regulating inflammatory processes [14].

There is a dilemma regarding the appropriate management of transplant candidates who have been infected with the SARS-CoV-2 virus as there is insufficient evidence to support whether stem cell transplantation should be postponed or not. The European Society for Blood and Marrow Transplantation (EBMT) recommendations advise that deferral of the transplant procedure should be considered in patients who are PCR positive for SARS-CoV-2 infection [15]. However, they acknowledge that this decision should be individualised, taking into account the risk of progression of the underlying disease. They advise that patients with high risk disease who have tested positive for SARS-CoV-2 should have their transplant procedure deferred for a minimum of 14 days and that they should be asymptomatic and have had two negative PCR tests for the SARS-CoV-2 virus that have been taken at least 24 hours apart [15]. In keeping with this guidance, the transplant procedure was deferred by four weeks in the case described in this report. We proceeded once PCR negativity for SARS-CoV-2 infection was confirmed and the CT changes were resolving [15].

## 3. Conclusion

To the best of our knowledge, this is the first report focusing on bone marrow architectural changes in the context of SARS-CoV-2 infection, in addition to the feasibility of proceeding to a nonmyeloablative allogeneic stem cell transplant with post-transplant cyclophosphamide for an elderly individual with high-risk AML less than one month following SARS-CoV-2 infection. Our report describes how the virus might precipitate marked bone marrow pathology in patients predisposed to inflammatory bone marrow states ( e.g., postchemotherapy or in myelodysplasia). In view of the very limited number of patients and the heterogeneity of haematological diagnoses of the patients described in this report, further multicentre clinical studies are warranted to best evaluate the bone marrow changes induced by SARS-CoV-2 infection and to inform the optimal peritransplant management of patients with recent SARS-CoV-2 infection.

## Figures and Tables

**Table 1:** Characteristics of haematology patients with SARS-CoV-2 infection who had simultaneous bone marrow biopsy.

Patient	Age	Gender	Diagnosis	Stage of disease	Date of bone marrow biopsy compared to most recent COVID PCR positivity	Bone marrow findings	Full blood count	Coagulation results	Outcome
Case described	70	Female	AML	In CR1, after 2 cycles of induction chemotherapy (CPX-351)	After 8 days	Suppressed myelopoiesis. No excess of blasts. Hyperplastic erythropoiesis with stress-type arrangement. Young forms appear megaloblastic. Reduced megakaryocytes with atypia. Many foamy histiocytes	Hb 86 g/L, WCC 2.36 × 10^9^/L, neutrophils 1.10 × 10^9^/L, lymphocytes 0.74 × 10^9^/L, monocytes 0.43 × 10^9^/L, platelets 15 × 10^9^/L	PT NA, APTT NA, fibrinogen NA, D-dimer NA	Now COVID-19 negative and has engrafted post-allogeneic stem cell transplant
2	68	Female	Multiple myeloma	Diagnosis	N/A	Severely reduced megakaryocytes, erythropoiesis, and myelopoiesis. >90% blastoid plasma cells	Hb 76 g/L, WCC 7.88 × 10^9^/L, neutrophils 2.44 × 10^9^/L, lymphocytes 3.58 × 10^9^/L, monocytes 1.79 × 10^9^/L, platelets 129 × 10^9^/L	PT 16.2 seconds, APTT 28.8 seconds, fibrinogen NA, D-dimer NA	Now COVID-19 negative. Ongoing induction chemotherapy
2	68	Female	Multiple myeloma	Midthird cycle of chemotherapy	After 3 days	Globally reduced megakaryocytes with nonspecific atypia. Normal myelopoiesis with scattered hyperplastic areas. Normal erythropoiesis with scattered hyperplastic areas. Postnecrotic scarring with numerous histiocytes and a few clusters of histiocytes. Scattered residual blastoid plasma cells	Hb 89 g/L, WCC 4.2 × 109/L, neutrophils 2.99 × 109/L, lymphocytes 0.71 × 109/L, monocytes 0.47 × 109/L, platelets 13 × 109/L	PT 12.65 seconds, APTT 28.35 seconds, fibrinogen 4.00 g/L, D-dimer 2034 ng/ml	Now COVID-19 negative. Ongoing induction chemotherapy
3	47	Male	Aplastic anaemia	Diagnosis	After 1 day	Myelopoiesis almost completely absent. Erythropoiesis almost completely absent. Megakaryocytes completely absent. Scattered increase in mast cells, which are very granulated. Less than 5% bone marrow cellularity	Hb 56 g/L, WCC 3.98 × 10^9^/L, neutrophils 0.5 × 10^9^/L, lymphocytes 3.36 × 10^9^/L, monocytes 0.11 × 10^9^/L, platelets 23 × 10^9^/L	PT 12.7 seconds, APTT 27.8 seconds, fibrinogen NA, D-dimer NA	Now COVID-19 negative. Awaiting allogeneic stem cell transplant for very severe aplastic anaemia
4	88	Male	MDS with multilineage dysplasia	Diagnosis	After 2 days	Suppressed left shifted myelopoiesis. 1–2% blasts. Dysplastic, hyperplastic erythropoiesis. Increased megakaryocytes with numerous dysplastic micromegakaryocytes. Moderate excess of mast cells that are degranulated	Hb 78 g/L, WCC 3.7 × 10^9^/L, neutrophils 0.48 × 10^9^/L, lymphocytes 2.90 × 10^9^/L, monocytes 0.32 × 10^9^/L, platelets 32 × 10^9^/L	PT 14.6 seconds, APTT NA, fibrinogen NA, D-dimer NA	Now COVID-19 negative. Ongoing support with G-CSF and erythropoietin
5	79	Male	Primary myelofibrosis	Suspected disease progression	After 7 days	Suppressed myelopoiesis. 8% blasts. Dysplastic erythropoiesis. Increased megakaryocytes which look dysplastic, including micromegakaryocytes. Diffuse stromal fibrosis, Bauermeister 3+	Hb 70 g/L, WCC 3.21 × 10^9^/L, neutrophils 1.76 × 10^9^/L, lymphocytes 0.91 × 10^9^/L, monocytes 0.52 × 10^9^/L, platelets 56 × 10^9^/L	PT 15.0 seconds, APTT 29.9 seconds, fibrinogen 4.17 g/L, D-dimer 1519 ng/ml	COVID-19 negative on discharge. Died of progressive disease
6	41	Male	Nodular lymphocyte predominant Hodgkin lymphoma	3 months after BEAM autograft	Before 4 days	Normal myelopoiesis. Normal erythropoiesis. Reduced megakaryocytes but no dysplastic features. Extensive stromal oedema and expansion of loose pathological extracellular matrix. No pathological lymphoid infiltrate	Hb 123 g/L, WCC 3.36 × 10^9^/L, neutrophils 2.80 × 10^9^/L, lymphocytes 0.43 × 10^9^/L, monocytes 0.13 × 10^9^/L, platelets 17 × 10^9^/L	PT 15.8 seconds, APTT 26.8 seconds, fibrinogen 5.31 g/L, D-dimer 11380 ng/ml	Died of COVID-19
7	65	Female	AML with MDS-related changes	6 weeks after allogeneic stem cell transplant	Before 13 days	Chaotic myelopoiesis. Normal erythropoiesis. Increased megakaryocytes, which are mostly small and dysplastic. 30–40% blasts. Widespread stromal oedema	Hb 85 g/L, WCC 1.45 × 10^9^/L, neutrophils 0.61 × 10^9^/L, lymphocytes 0.5 × 10^9^/L, monocytes 0.28 × 10^9^/L, platelets 33 × 10^9^/L	PT NA, APTT NA, fibrinogen NA, D-dimer NA	Died of disease relapse

## Data Availability

No data were used in this study.

## Abbreviations

AML: Acute myeloid leukaemia
CR: Complete remission
CPX-351: Liposomal daunorubicin/cytarabine
Hb: Haemoglobin
WCC: White cell count
PT: Prothrombin time
PTT: Partial thromboplastin time
COVID-19: Corona virus disease 2019
MDS: Myelodysplasia
BEAM: Carmustine/etoposide/cytarabine/melphalan.
